# Paroxysmal extreme pain disorder in family with c.3892G > T (p.Val1298Phe) in the *SCN9A* gene mutation – case report

**DOI:** 10.1186/s12883-020-01770-9

**Published:** 2020-05-13

**Authors:** Adam Stępień, Daria Sałacińska, Jacek Staszewski, Marta Durka-Kęsy, Jan Dobrogowski

**Affiliations:** 1 Department of Neurology, Military Institute of Medicine, 128 Szaserów Street, 04-141 Warsaw, Poland; 2 Department of Pain Research and Treatment, Chair of Anesthesiology and Intensive Therapy Jagiellonian University College of Medicine, Krakow, Poland

**Keywords:** Paroxysmal extreme pain disorder, *SCN9A* gene mutation

## Abstract

**Background:**

To describe the clinical phenotype of paroxysmal extreme pain disorder, an autosomal dominant condition in four members in one family with the mutation NM_002977.3:c.3892G > T (p.Val1298Phe) in the *SCN9A* gene. Clinical examinations and details from members of one Polish family were collected, including age at onset, features of attacks, problems between attacks, investigational results, treatments tried, and evolution over time.

**Case presentation:**

Twenty two individuals from this family with paroxysmal extreme pain disorder were identified. Seven of them presented clinical manifestation of paroxysmal extreme pain disorder, of which and in four were identified missens mutations in the SCN9A gene (NM_002977.3:c.3892G > T). The onset of the disorder took place in the neonatal period or infancy and persists throughout life. Autonomic manifestations predominate with extreme pain, skin flushing and harlequin colour change were observed in all. Attacks of excruciating deep burning pain often appear in the rectal, or jaw areas, but also diffuse in the body. Attacks are triggered by factors such as: defecation, eating, pressure and emotion. Carbamazepine and other antiepileptic drugs were only partly effective in almost all, but the response was incomplete.

**Conclusions:**

Paroxysmal extreme pain disorder is a hereditary sodium channelopathy with pain and an autonomic nervous system dysfunction. Paroxysmal extreme pain disorder is rare, so far only 500 cases of both women and men have been described in world literature.

## Background

Paroxysmal extreme pain disorder (PEPD) is a genetically conditioned autosomally dominantly inherited chronic disease characterized by attacks of severe pain located in various areas of the body combined with skin flashing. The mutation refers to the *SCN9A* gene encoding proteins forming the Na_V_1.7 sodium channel in sympathetic ganglia neurons [1]. The disorder is rare, so far only 500 cases of both women and men have been described in world literature [2, 3].

Clinical symptoms are characterized by attacks of rapidly developing burning, lancinating pain in the rectal, ocular and mandibular areas with skin flashing in a harlequin pattern. The pain lasts from a few seconds to several hours. It may be accompanied by apnoea, high blood pressure, asystole or epileptic seizures. The first symptoms of the disease usually appear during infancy. Most often their appearance is related to provocative factors such as: defecation, eating, taking medications, micturition, gynaecological examination, rectal examination, stress or even touch [1, 2, 4]. In the presented family on the basis of the typical features of the attacks, a diagnosis of paroxysmal extreme pain disorder (PEPD) was made and confirmed by molecular genetics.

**Fig. 1:**
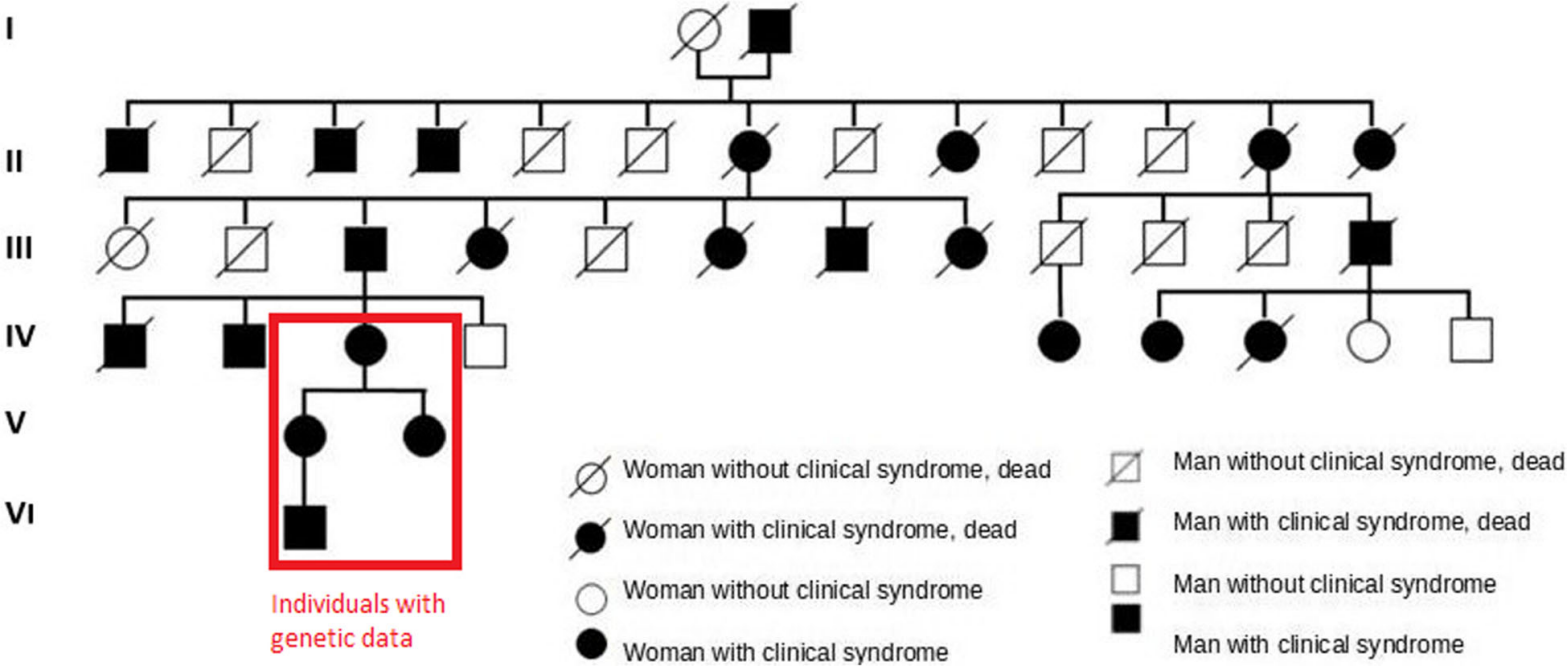
A six-generation pedigree displaying affected members of the family

Clinical examinations and details from four members of one Polish family were collected, including age at onset, features of attacks, problems between attacks, investigational results, treatments tried, and evolution over time. Twenty two individuals from this family with paroxysmal extreme pain disorder were identified (Fig.1).

## Case presentation

The 44-year-old patient (patient IV.3) applied for the consultation because of many years of recurrent attacks of severe pain of a tearing character and significant intensity located in different parts of the body. Initially, the pain attacks were located only in the perineum area; they appeared suddenly and lasted from a few seconds to several minutes. They were preceded by a triggering factor, such as: irritation of the perineum area (e.g. during defecation), pressure, scratch or a stressful situation. The pain was accompanied by additional symptoms, which varied depending on the patient’s age. The patient reported that the first episode had occurred at the age of about 8 months, during passing stool, when the cry had been accompanied by bending the body into a “cradle” and apnoea. The patient remembers the attacks from about the age of 7–8. At that time, the pain was accompanied by shortness of breath, flushing of half or parts of the body (e.g. half the chest as well as ocular and submaxillary regions) and a hot feeling on the side of flushing. The pain was most often felt to appear in superficial tissues, but could also be felt in deep tissues during severe attacks. At a later age, the location of pain during the attacks changed - there were headaches accompanied by tearing or abdominal pain. No abnormalities were detected in the additional examinations, including cerebral MRI, EEG and ECG. The patient took carbamazepine in the past, which turned out to be ineffective. Currently, a partial improvement and an alleviation of symptoms have been achieved using topiramate and pregabalin. Despite these problems, the patient’s lifestyle is normal and there is no experience of any other additional disability.

**Fig. 2:**
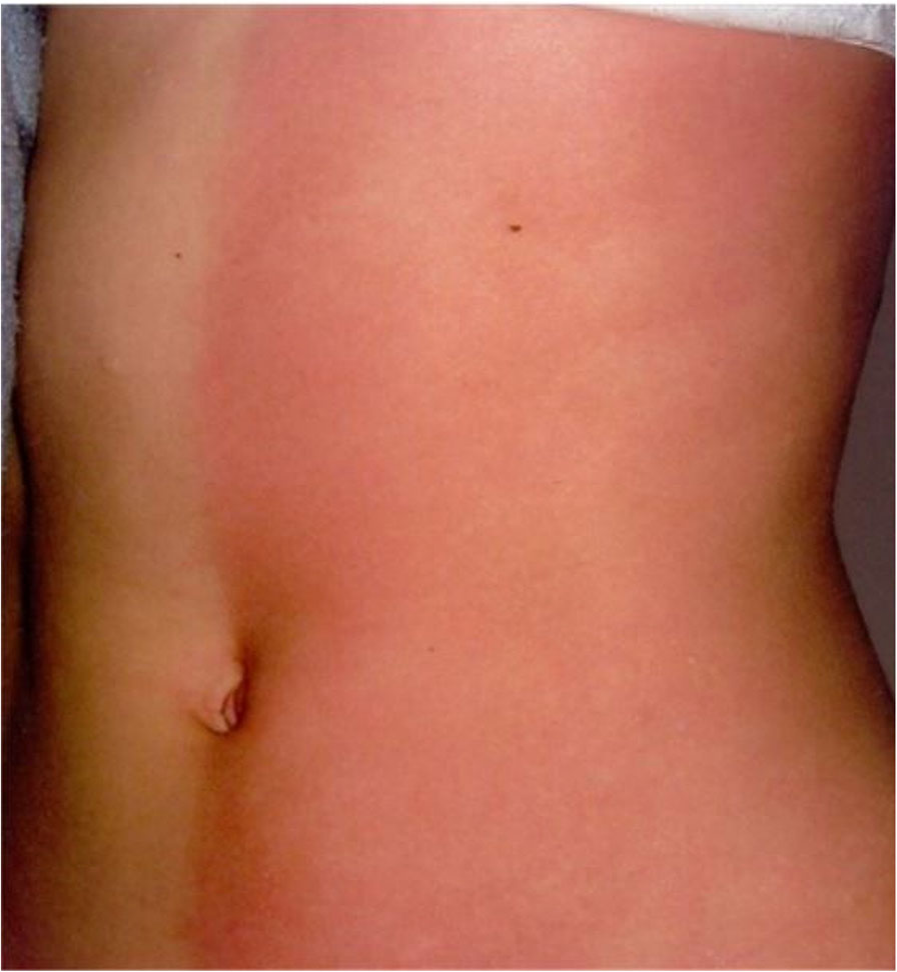
Harlequin flushing in family members during episodes of paroxysmal extreme pain disorder

**Fig. 3:**
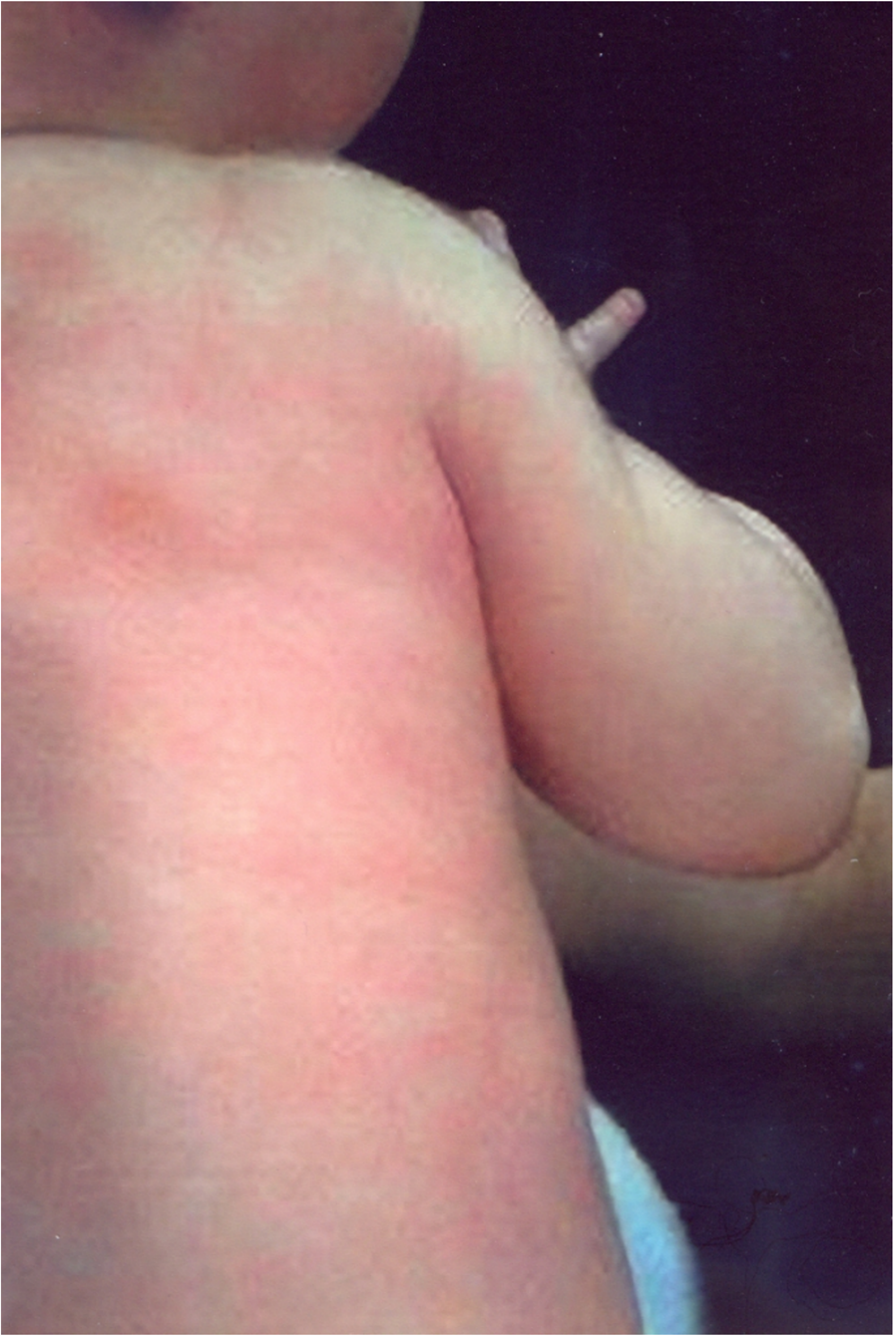
Harlequin flushing in family members during episodes of paroxysmal extreme pain disorder

**Fig. 4:**
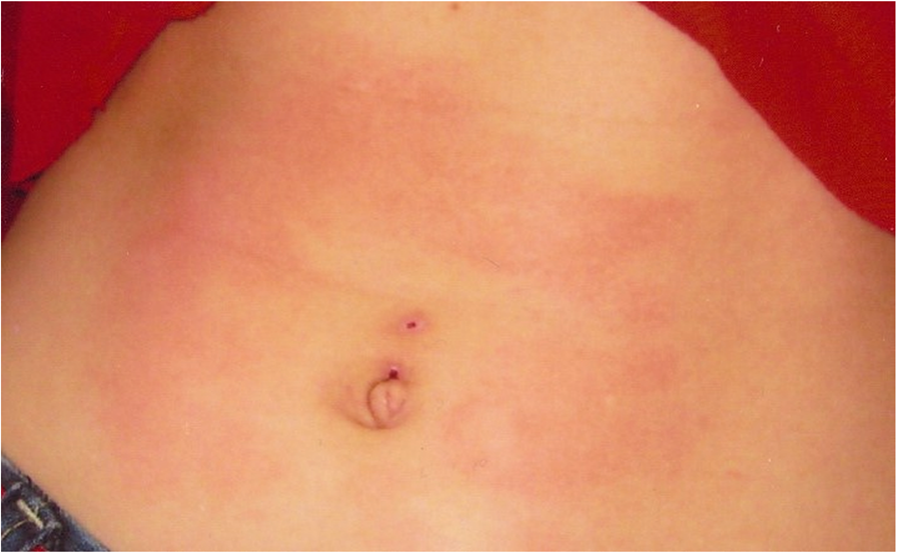
Harlequin flushing in family members during episodes of paroxysmal extreme pain disorder

**Fig. 5:**
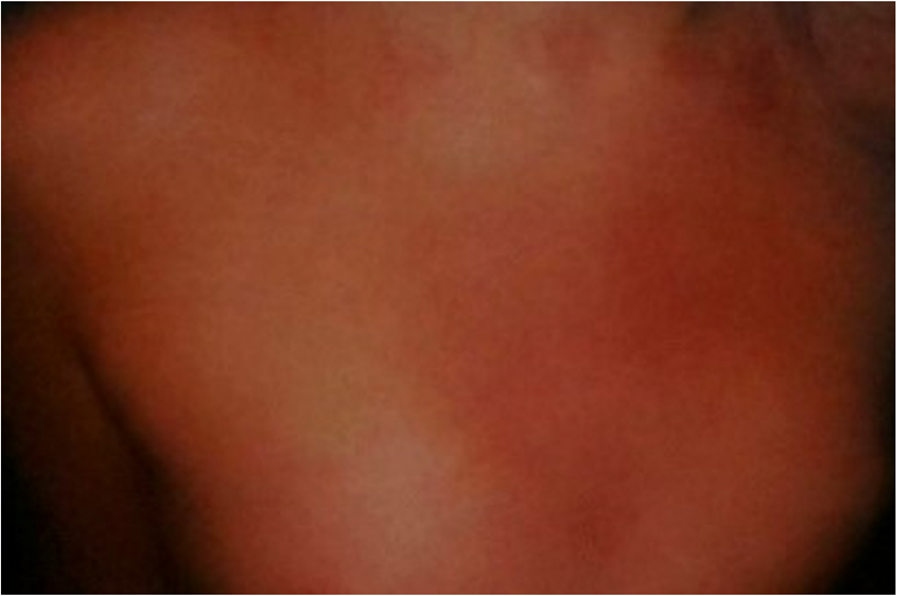
Harlequin flushing in family members during episodes of paroxysmal extreme pain disorder

Similar symptoms occur in the 20-year-old (patient V.2) and 25-year-old (patient V.3) daughters of the patient. They were repeatedly hospitalized in their childhood due to the presence of apnoeas, convulsions, contractures of the limbs or fainting. The picture of these attacks also changed with their age. In the older daughter the first episode occurred at the age of 2.5 months, during a bath, in the form of body flushing, apnoea with spontaneous breath back, vociferous crying, fatigue and flaccidity of the muscles. During the first hospitalization, the pain attack occurred during the rectal examination in the form of several deep breaths followed by apnoea and spontaneous breath back, and then drowsiness lasting about 1–2 min, body flashing and the increase of the right lower limb warmth were observed. In the early childhood, the episodes of pain were accompanied by loss of consciousness, with urinating and biting the tongue. The pain attacks also occurred in the night in the form of severe headaches with half the face flashing, and eye tearing on the side of flashing. After the attack, which regressed spontaneously, intense sweating, mainly in the head occurred. Periodically, also the attacks of abdominal pain or headaches with flashing of the skin layers were observed (Figs. 2, 3, 4, 5). The attacks were preceded by a painful or emotional stimulus. During the teenage period, fainting dominated and later contractures of the upper limbs joined. Between attacks, the patient’s neurological development was normal.

During attacks, hypersensitivity of the skin at the side of redness and increased sweating were observed in all patients (IV.3, V.2, V.3). Between attacks, the assessment of sensory and autonomic functions was correct. No changes were found in neurological examination either. No abnormalities were revealed in the repeatedly performed haematological, biochemical investigations as well as head imaging examinations, including MRI and an electroencephalographic examination. So far, none of these patients has been treated chronically for another reason.

The older daughter’s four-year old son (patient VI.1) also suffers from excruciating pain and flushing upon benign mechanical or heat stimuli mostly in rectal or abdomen regions. A typical episode appeared during the first few days of his life, with paroxysmal painful events that started with tonic contraction of the whole body followed by erythematous harlequin-type colour changes. After the attack, which regressed spontaneously, the infant experienced intense sweating.

The genetic test was performed in four family members. All four tests confirmed the heterozygous mutation NM_002977.3:c.3892G > T (p.Val1298Phe) in the *SCN9A* gene (Fig.1). Genetic test was made with Sanger method.

## Discussion and conclusions

Mutations in the *SCN9A* gene are the cause of a heterogeneous channelopathy pain syndrome including small nerve fiber neuropathy, inherited erythromelalgia and PEPD. In the presented family with PEPD NM_002977.3:c.3892G > T (p.Val1298Phe) missens mutation in the *SCN9A* gene was detected. This mutation is reported in ClinVar Database (variation ID 6358; rs121908912) as patogenic in PEPD.

Recurrent episodes of pain in all members of the family affected different body parts and were accompanied by autonomic manifestations, such as: skin flushing and swelling of the skin, which occur in a harlequin pattern.

The recognition of PEPD is based on the diagnosis of a characteristic set of symptoms, a course of the disease and a genetic test. In the initial period it can be difficult and requires numerous diagnostic tests that exclude other than the genetic mutation background of the disease. The first description of the disease was made by Hayden and Grossman in 1959 [5]. It was then noted that patients often experienced attacks of very strong burning pain in the rectal area during defecation. In later reports, it has been observed that pain attacks with skin flushing can also be located on the trunk, head or limbs, and the trigger can be touch or stress. The first symptoms of the disease may appear immediately after a childbirth in the neonatal period. The disease is associated with the occurrence of “mutations” in the *SCN9A* gene located in the 2q24.3 chromosome coding for alpha subunit of the potential-dependent Na_V_1.7 sodium channels. These channels are mainly expressed in peripheral somatic and visceral sensory nerves, nociceptoras, dorsal roots of the spinal cord, trigeminal ganglion, olfactory cells and sympathetic ganglion [2]. The relationship of Na_V_1.7 with the feeling of pain was confirmed, and the presence of a homozygous *SCN9A* gene mutation causing loss of the channel function is associated with a complete inability to feel pain (congenital pain insensitivity syndrome) [6]. The mutation triggering PEPD causes the creation of Na_V_1.7 channels with a changed function, which interferes with the inactivation process. The extension of the phase of rapid inactivation of the channels, generating of the constant sodium current and the increased excitability of neurons occur [7]. Due to literature, authors of molecular study of *SCN9A* gene in PEPD were identified eight mutations of *SCN9A* in these disease [1]. In these mutations was also identified mutation which was found in described family. At present, according to the ClinVar database and literature, ten PEPD-related mutations are known [2]. Eight of them are pathogenic for PEPD. Clinically defined PEPD do not always harbour the *SCN9A* gene mutation [8]. For example, erythema and burning pain in the lower parts of the body, more classically a primary erythromelalgia description, is harbouring the M1627K mutation of *SCN9A* gene [9]. As far as the diversity of symptoms and different genetic origins are concerned, a clinical diagnosis of PEPD might encompass different entities. In the described family, there is a consensus between the *SCN9A* gene mutation and the phenotype.

Symptoms of PEPD occur throughout the entire life of an individuals with PEPD [8]. However, the frequency and nature of attacks vary with age, with the highest intensity during infancy and early childhood. Tonic nonepileptic seizures are more characteristic of infancy and early childhood, but may also occur in adults. These are accompanied by apnoea, flushing, bradycardia and skin changes of the harlequin type, without crying. In a later age, an attack changes its image slightly, starting from an inconsolable screaming that changes into apnoea, pallor and stiffness. Such an episode lasts from a few seconds to a few minutes and then the breath comes back spontaneously. After the attack, there is an impaired contact and flaccid for a few minutes [8]. They may be accompanied by periods of extended asystole requiring resuscitation. So far, no individual’s death has been recorded as directly caused by a sudden circulatory arrest in the course of a pain attack [3]. During late childhood and in adults, ocular and mandibular attacks may occur more frequently. The number of attacks caused by irritation of the rectal area is reduced by the use of methods to prevent constipation and a learned way of defecation [8]. Between attacks, individuals complain of constipations, which may be caused by fear of triggering an attack. Till now, there has been no study assessing the impact of PEPD on the psychic and psychological status of subjects, however, cases of depression and drug abuse are observed in these group. The cognitive and intellectual level of individuals with PEPD is similar to the general population [7, 8].

A genetic test in PEDS is indicated when there is:

● pain of a similar nature of symptoms in > 2 family members.

● pain of unknown aetiology at an early age.

● unusual picture of the disease.

● another genetic disorder that may be contributing to pain [2].

Currently, we do not have any causal treatment for paroxysmal extreme pain disorder. Treatment is based on the use of sodium channel inhibitors. Antiepileptic drugs, tricyclic antidepressants and inhibitors of the reabsorption of serotonin and noradrenaline are used. The drug of choice in PEPD is carbamazepine [8]. It is observed to be partially effective in reducing the number and severity of attacks in the majority of subjects with PEPD, and in individual cases even their complete elimination. It seems that other antiepileptic drugs are partially effective in some cases, e.g. gabapentin, topiramate or lamotrigine. The use of amitriptyline, clonidine and intravenous lidocaine does not bring the desired effect. Opioid drugs are not recommended due to both their ineffectiveness and the possibility of increased constipation. In children, in cases of the strongest attacks, an improvement is obtained after using a 1: 1 mixture of nitrous oxide and oxygen [8, 10]. In the literature the beneficial effects of pelvic floor muscle exercises and the prevention of constipation are described [2]. In circumscribed cases only a very limited range response for antiepileptic drugs and tricyclic antidepressants was observed.

## Data Availability

The datasets used and analysed during the current study are available from the corresponding author on reasonable request.

## Abbreviations

PEPD: Paroxysmal extreme pain disorder
MRI: Magnetic resonance imaging
EEG: Electroencephalography
ECG: Electrocardiography
